# Underwater Noise from a Wave Energy Converter Is Unlikely to Affect Marine Mammals

**DOI:** 10.1371/journal.pone.0132391

**Published:** 2015-07-06

**Authors:** Jakob Tougaard

**Affiliations:** Department of Bioscience, Aarhus University, DK-4000, Roskilde, Denmark; University of Pavia, ITALY

## Abstract

Underwater noise was recorded from the Wavestar wave energy converter; a full-scale hydraulic point absorber, placed on a jack-up rig on the Danish North Sea coast. Noise was recorded 25 m from the converter with an autonomous recording unit (10 Hz to 20 kHz bandwidth). Median sound pressure levels (L_eq_) in third-octave bands during operation of the converter were 106–109 dB re. 1 μPa in the range 125–250 Hz, 1–2 dB above ambient noise levels (statistically significant). Outside the range 125–250 Hz the noise from the converter was undetectable above the ambient noise. During start and stop of the converter a more powerful tone at 150 Hz (sound pressure level (L_eq_) 121–125 dB re 1 μPa) was easily detectable. This tone likely originated from the hydraulic pump which was used to lower the absorbers into the water and lift them out of the water at shutdown. Noise levels from the operating wave converter were so low that they would barely be audible to marine mammals and the likelihood of negative impact from the noise appears minimal. A likely explanation for the low noise emissions is the construction of the converter where all moving parts, except for the absorbers themselves, are placed above water on a jack-up rig. The results may thus not be directly transferable to other wave converter designs but do demonstrate that it is possible to harness wave energy without noise pollution to the marine environment.

## Introduction

There is increasing focus on underwater noise emitted from offshore installations and the possible detrimental effects this noise can have on marine mammals. A recent addition to the anthropogenic sources of underwater noise is offshore wave energy converters. Although the technology of wave energy converters is still in its infancy, there are several test systems currently in operation and a substantial capacity can be expected to be installed in coming years in coastal and offshore waters, as European countries strive to fulfil the common goal of reducing dependence on fossil fuels in energy production[1]. These wave energy converters are expected to produce underwater noise during operation[2, 3] and naturally, this has raised concern about possible impact on fish and marine mammals[4] in the same way as has been the case for offshore wind energy and other human activities on the ocean[5, 6].

Very limited information is available about the levels and characteristics of underwater noise emitted from wave energy converters. There are several, principally very different ways to realise a wave energy converter and frequency spectrum and levels of noise generated by a converter is likely to depend on the particular implementation. The three principal types of converters are attenuators, which are oriented parallel to the direction of wave propagation and capture the energy by flexing of the converter; point absorbers, which translate wave energy to an up-and-down motion used to generate hydraulic or air pressure; and terminators, which are oriented perpendicular to the direction of wave propagation[1]. One study[3] measured noise around a 1/7^th^ scale point absorber (SeaRay prototype) and found frequency modulated signals with peak frequency around 100 Hz and modulated with a rate corresponding to the wave period and generator shaft speed. Although no recordings were made of ambient noise without the converter in operation, which would have allowed a direct comparison, it appears likely that the frequency modulated noise originated from the converter. Noise levels were low, with mean broadband (60 Hz-30 kHz) sound pressure levels (L_eq_) around 122 dB re 1 μPa. Another study[7] measured noise from an experimental full scale point absorber (Lysekil, Sweden). This point absorber produced very prominent impulsive sounds, with energy up to at least 20 kHz, but with most energy below 1 kHz, at average levels of 129 dB re. 1 μPa measured about 20 m from the absorber. The source of the impulsive sounds was, however, found to be the linear converter hitting the end stop springs. The loud noise can therefore be seen more as the result of a design error or maladjustment of the absorber, rather than being representative of the noise from a properly operating wave energy converter.

**Fig 1:**
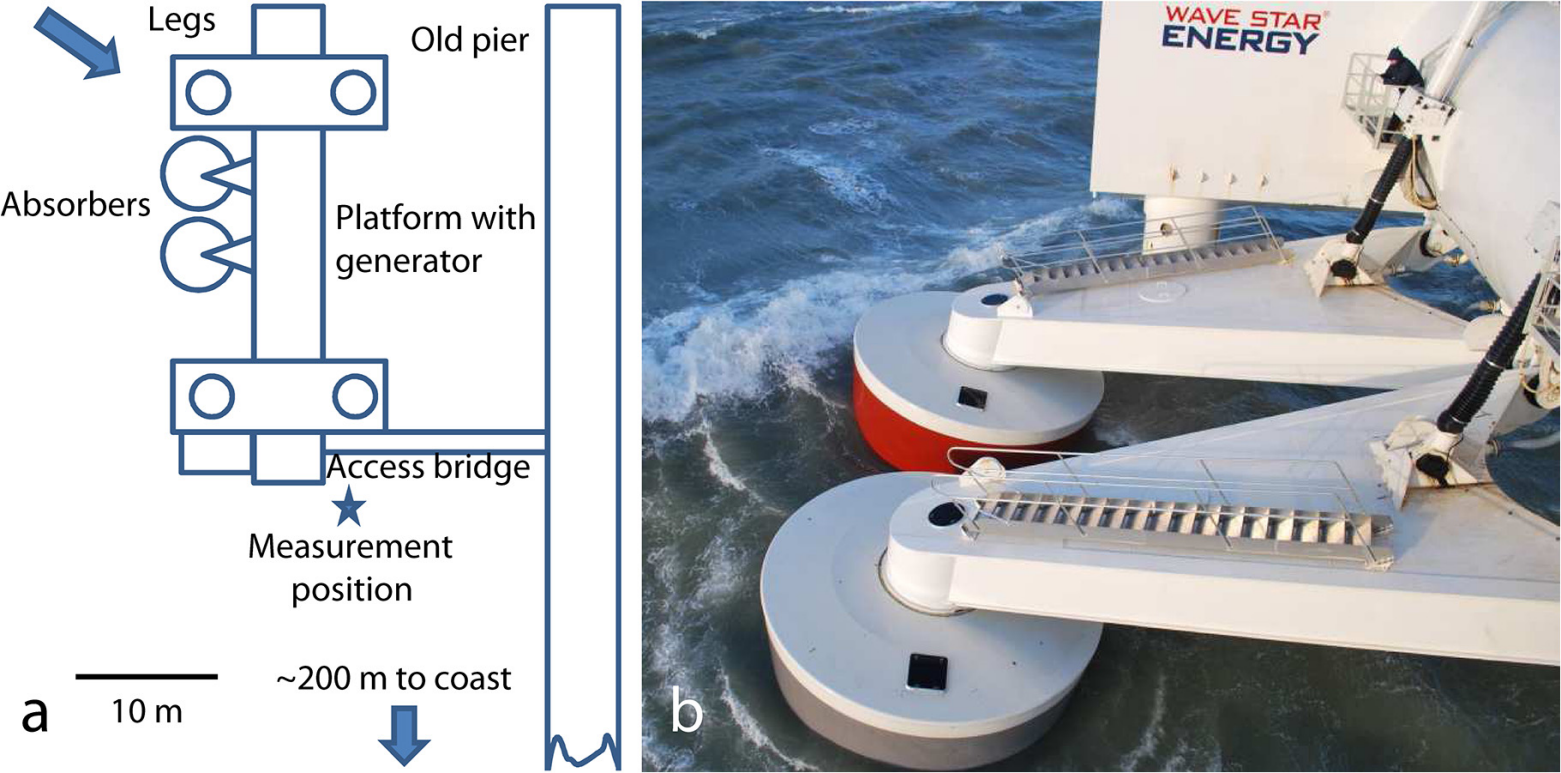
The Wavestar wave energy converter, located on the Danish North Sea coast. a) Schematic drawing of the wave energy converter, placed at the end of a stone pier, about 200 m from the coast. Recordings were made at the position indicated by the star. Blue arrow indicates wave direction during measurements. b) Photo of the two absorbers during operation. Note the person next to the hydraulic piston for scale.

An alternative realisation of a point absorber is the Wavestar[8]. In contrast to the 1/7^th^ scale SeaRay, the Wavestar is a full-scale test and demonstration converter. The Wavestar consists of a number of absorbers (two in the present implementation), hinged onto a four-legged jack-up platform (Fig 1). During operation the independently operating absorbers float semi-submerged in the water and wave-generated up-and-down motion is converted into hydraulic pressure by means of pistons connected to the arms of the absorbers. The hydraulic pressure generated in the pistons is rectified and drives the generator. The jack-up design allows continuous adjustment to the tide and offers storm protection. During a storm the entire platform can be jacked up to a safe height above the waves. When absorbers are taken out of operation, due to service or storm protection, they are raised completely out of the water by the hydraulic pistons.

The nominal capacity of the Wavestar with two absorbers is 110 kW. Because of the larger size compared to the SeaRay higher noise levels may be expected, but this may be counteracted by the fact that the hydraulic system and generator is located above water in the Wavestar and not inside the absorber as in the SeaRay. This limits the surface which is in contact with the water to the legs and the absorbers themselves.

## Measurements

Underwater noise was recorded on October 24^th^ 2012 at the Wavestar wave energy converter located at Hastholm, Denmark (57°7.73´N, 8°37.23´E). An autonomous datalogger (DSG-marine, Loggerhead Instruments, Sarasota, Florida) with a HTI-96min hydrophone (sensitivity -186 dB re. 1 V/μPa) connected to a 35 kHz low-pass filter (3-pole Butterworth) was used for recording. Noise was continuously digitized with 16 bit resolution at 80 ksamples/s, low-pass filtered, decimated to 40 ksamples/s and stored to an SDHC memory card. The datalogger was attached to an anchor and moored in 7 meter deep water, about 2 m above the seabed with a hard plastic ball as flotation. Deployment was 25 m from the center of the nearest absorber and 17 m from the stone pier (Fig 1). Weather during recordings were NW-wind (305°), 8.6 m/s (10 min average), significant wave height 1.9 m, wave period 4.2 s. The converter was operating close to maximum power output. Access to the Wavestar facility was granted by Wavestar A/S and passive acoustic measurements of this type do not require a permit under Danish Law.

A 57 minute sequence of noise was analyzed in Matlab (Mathworks, R2010b). This sequence contained recordings of ambient noise, the converter in full operation and start and stop of the converter. The recording was subdivided into two sequences of the converter running (31 minutes in total), two sequences of the converter stopped (including all hydraulic pumps etc.) and absorbers lifted out of the water (14 minutes in total), two sequences of the shut-down procedure, where absorbers one by one were emptied for ballast water and lifted into resting position (9 minutes in total) and one sequence of start-up procedure, simultaneous lowering of both absorbers, followed by filling with ballast water (3 minutes).

## Results

**Fig 2:**
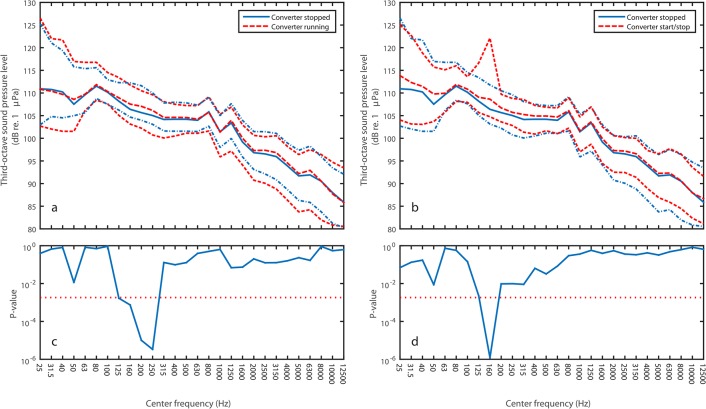
Frequency spectra of noise recorded close to the wave energy converter with the converter stopped and in different stages of operation. Noise levels are expressed as median (L_50_) and upper and lower 5% percentile (L_5_ and L_95_) sound pressure levels in third-octave bands. a) Comparison between ambient noise spectrum (converter stopped, solid blue line; percentiles: dot-dashed blue lines) and noise spectrum recorded with the wave energy converter running (red dashed line; percentiles: red fine dashed lines). b) Comparison between ambient noise spectrum (converter stopped, solid blue line; percentiles: dot-dashed blue lines) and noise spectrum recorded during start and stop of the converter (red dashed line; percentiles: red fine dashed lines). Self-noise of the recorder was below 80 dB re. 1 μPa in all third-octave bands. c) Results of pair-wise Mann-Whitney tests (p-value) for all third-octave bands comparing converter running to ambient noise. Broken line indicates 5% significance level, adjusted for multiple comparisons (Dunn-Sidak correction). d) Same as c), but comparing start and stop of converter to ambient noise.

Median broad band (10 Hz–20 kHz) sound pressure level (L_eq_) was 123 dB re. 1 μPa, irrespective of status of the wave energy converter (stopped, running or starting/stopping). The recorded sequence was subdivided into 10 s periods (186, 84 and 72 periods with the converter running, stopped and starting/stopping, respectively) and one-third octave levels were computed for each segment by means of the Matlab-function filtbank (Christophe Couvreur, Faculte Polytechnique de Mons, Belgium). Median (L_50_) and upper and lower 5% percentiles (L_5_ and L_95_) of third-octave sound pressure levels are shown in Fig 2A and 2B for the three states of the converter (running, stopped and start/stop procedure). Levels were compared pairwise within each 1/3 octave band by Mann-Whitney’s U-test to test for systematic differences among the three states (Fig 2C and 2D). A significance level *α* of 5% was used, but adjusted for multiple comparisons by the method of Dunn-Sidak, by which an adjusted value for *α* is obtained: *α*′ = 1 − 1(1 − *α*)^1/*n*^, where *n* is the number of pairwise comparisons (third-octave bands). An example of the noise from the running converter is found in the supplementary material as S1 Audio.

The most pronounced peak in the third-octave was in the 160 Hz band during start and stop of the converter (Fig 2B and 2D). Closer inspection revealed this to be a pure tone signal at 150 Hz about 25–30 s in duration, followed by a linear downsweep in frequency. The sound pressure level (L_eq_) of the tone was 121–125 dB re 1 μPa. Listening to the sound recording clearly identified the source of this noise as the hydraulic pump responsible for lifting and lowering the absorbers in and out of the water. The hydraulic pump is located above the water line inside the jack-up and the noise thus most likely reached the water through vibrations of the legs of the jack-up. An example of the pump noise is found in the supplementary material as S2 Audio.

Less pronounced, but still statistically significant differences were seen in the bands 125, 160, 200 and 250 Hz when operation and ambient were compared (Fig 2A and 2C). No statistically significant noise above ambient could be detected above the 250 Hz band (Fig 2C and 2D). The absolute increase in noise above ambient was very small. L_50_ third-octave levels in the four bands with the converter running were thus only 1–2 dB above ambient L_50_ levels.

All in all, the noise recorded from the converter at a distance of 25 m to the absorber was barely detectable above the ambient noise in the frequency range 125–250 Hz. As the noise during operation has energy in the same frequency range as the noise during start and stop the hydraulic system is suggested as a likely source of the noise during operation as this was clearly the source during start and stop.

## Discussion

**Fig 3:**
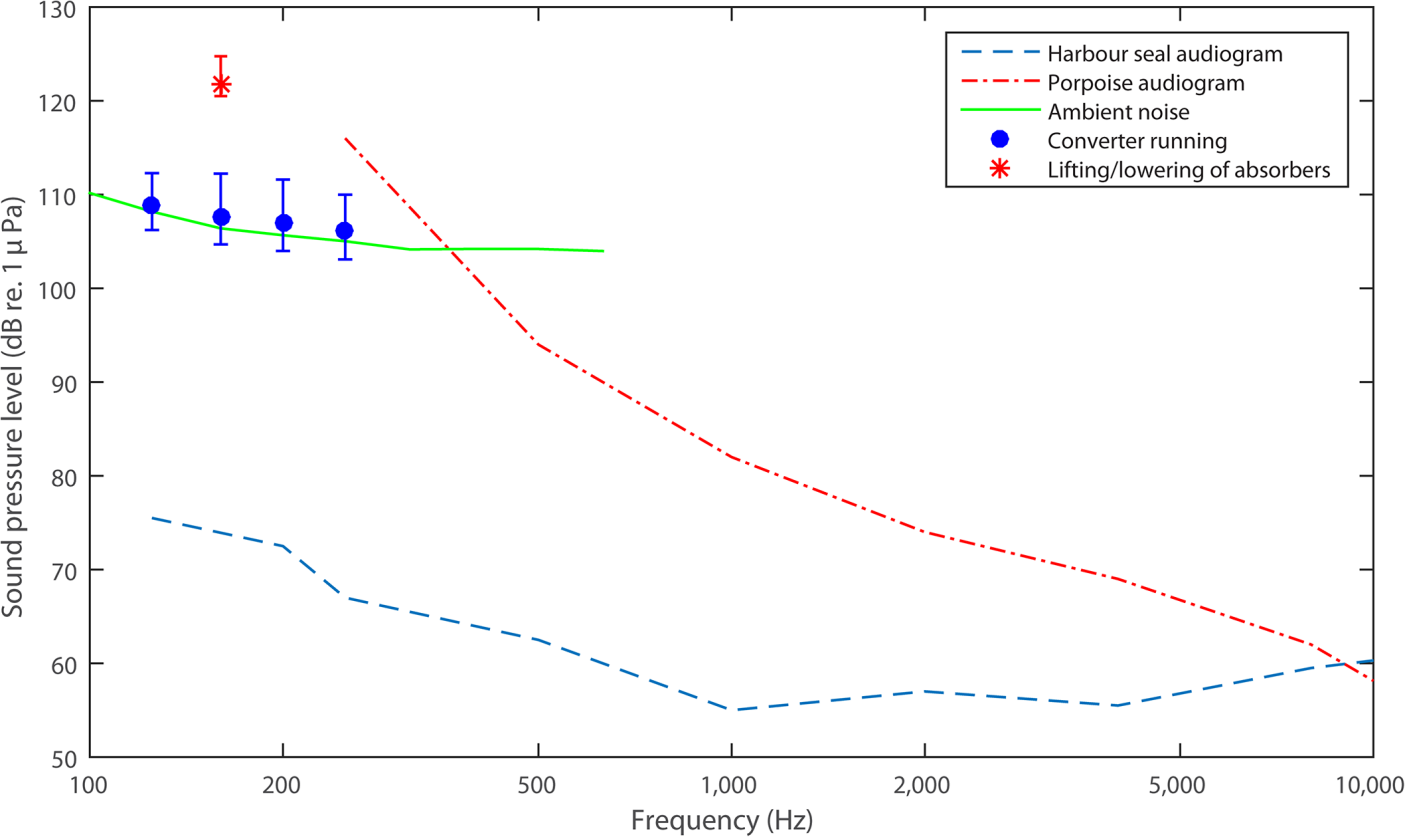
Audibility of converter noise to harbour seals and harbour porpoises. Audiograms of harbour seal and harbour porpoise are compared to median (L_50_) third-octave levels of noise from the wave energy converter in the bands where levels were statistically significant above ambient noise. Error bars indicate upper and lower 5% percentiles (L_5_ and L_95_). Solid line indicates median ambient noise level (L_50_). Indicated is also the median sound pressure level of the tone generated by the hydraulic pump during lifting and lowering of the absorbers (star). Error bars indicate minimum and maximum levels recorded. Audiograms from[9, 10].

The noise recorded from the wave energy converter was barely detectable above ambient noise and in order to discuss possible negative effects of the converter noise on marine mammals it is relevant first to ask whether they are able to hear the noise above ambient at all. Third octave band levels in the bands 125–250 Hz are compared to ambient noise and audiograms for harbour seals[9] and harbour porpoises[10], the two most common marine mammals in the North Sea (Fig 3). Harbour seals have good low frequency hearing and third-octave levels of the converter noise are well above the hearing threshold. Harbour seals are thus expected to be able to hear the converter noise, although the elevation in noise levels is so low (1–2 dB) that it is likely to be close to inaudible even at the close range where recordings were obtained. The exception is the noise generated by the hydraulic pump during lifting and lowering of the absorbers. This noise was 20–25 dB above ambient and should have been clearly audible to seals.

In contrast to seals, harbour porpoises have poor low frequency hearing and it seems unlikely that the converter noise would have been audible to porpoises. Even the noise from the hydraulic pump is below the hearing threshold for porpoises and unlikely to have been audible.

Ambient noise levels in the recordings were comparatively high, which could be due to the location of the converter: close to shore and next to a stone pier. Ambient noise levels could thus be expected to be lower in a production installation located offshore. In addition, the combined noise from up to 20 absorbers per unit in a production installation[11] may also be higher than the measured noise for two absorbers. At the very most, if noise from the absorbers is independent and added completely, this would amount to an additional 10 dB ($10\;{\text{log}}_{10}\frac{20}{2}$), but in reality considerably less, as noise from the more distant absorbers would contribute very little. However, this could mean that the audibility of the noise for seals could be higher from an offshore production unit, whereas the absolute levels of the noise would still be so low that harbour porpoises would still not be able to hear the noise even under more quiet conditions and with more absorbers.

It is of interest to compare the noise levels recorded from the converter to recordings from offshore wind turbines[6, 12, 13]. This comparison is illuminating for two reasons. The first is that it has been suggested to deploy the Wavestar converter in a star-shaped configuration around existing offshore wind turbines[11], which means that the noise from the wave energy converter will be added to the contribution already present from the wind turbine. The second reason a comparison is relevant is that despite several studies on the effects of offshore wind farms on porpoises[14] no detrimental effect of the noise has so far been documented. Underwater noise from wind turbines has energy in the same frequency range as the Wavestar converter (below 500 Hz), but with higher sound pressure levels. Betke[12] measured noise from an offshore wind turbine at Horns Reef in the North Sea under partial loading at a wind speed of 8.6 m/s, comparable to the conditions under which the Wavestar noise was recorded. Third-octave levels (L_eq_) of the wind turbine noise in the range 100–315 Hz were 95–105 dB re 1 μPa, i.e. roughly comparable to the wave energy converter noise, but measured at a distance of 87 m from the turbine in contrast to the 25 m for the wave energy converter. Thus, even under the most favourable sound transmission conditions for the wave energy converter noise, it is not likely to add substantially to the noise already present from the wind turbine. This, however, would not be the case all the time, as there is usually a time lag between wind and waves[15], such that in decreasing winds the wave energy converter may continue to generate power for several hours after the wind turbines have stopped[16].

Wave energy converters come in many different designs and work according to a number of different principles. Other types of converters could be expected to be noisier, perhaps also to generate noise at other frequencies than those reported from the Wavestar. The present conclusion, that noise levels from the Wavestar point absorber are unlikely to affect seals and porpoises, thus cannot be taken as evidence that all wave energy converters are unproblematic with respect to noise. Nevertheless, the results clearly demonstrate that it is possible to harvest wave energy in a way which does not add substantially to the increasing levels of anthropogenic noise in the ocean.

## Supporting Information

S1 Audio Sample of the noise recorded from the running converter. (WAV)

S2 Audio Sample of the noise recorded from the hydraulic pump during lowering of the absorbers. (WAV)
